# The EORTC QLQ-CR29 quality of life questionnaire for colorectal cancer: validation of the Dutch version

**DOI:** 10.1007/s11136-015-1210-5

**Published:** 2015-12-28

**Authors:** A. M. Stiggelbout, M. Kunneman, M. C. M. Baas-Thijssen, P. A. Neijenhuis, A. K. Loor, S. Jägers, R. Vree, C. A. M. Marijnen, A. H. Pieterse

**Affiliations:** Department of Medical Decision Making, Leiden University Medical Center, Leiden, The Netherlands; Department of Surgery, Alrijne Hospital Leiderdorp (Location Rijnland), Leiderdorp, The Netherlands; Department of Surgery, Leiden University Medical Center, Leiden, The Netherlands; Clinical Trial Center, Erasmus Medical Center Cancer Center, Rotterdam, The Netherlands; Department of Surgery, Alrijne Hospital Leiden (Location Diaconessen), Leiden, The Netherlands; Department of Radiotherapy, Leiden University Medical Center, Leiden, The Netherlands

**Keywords:** Health-related quality of life, Colorectal cancer, QLQ-CR29, Validation

## Abstract

**Purpose:**

To validate the Dutch version of the EORTC QLQ-CR29 quality of life questionnaire for colorectal cancer.

**Methods:**

We translated and pilot-tested the original questionnaire in the Netherlands, following EORTC guidelines. We assessed factor structure, reliability and construct validity in different samples of patients from four hospitals.

**Results:**

Of 296 patients, 236 (80 %) returned the questionnaire, and 27 out of 48 patients returned the retest questionnaire. In addition to the original three scales, we found a reliable bowel functioning scale (*α* = 0.80), reducing the number of individual items by five. Two of the other scales had sufficient to good reliability (urinary frequency, *α* = 0.71, original *α* = 0.75, body image *α* = 0.80, original *α* = 0.84), the third, blood and mucus in stool, only moderate (*α* = 0.56, original *α* = 0.69). Item functioning was sufficient to excellent for all but two items (urinary incontinence and dysuria). Construct validity was similar to that in earlier studies.

**Conclusion:**

We found a very satisfactory scale for bowel problems, in patients both with and without stoma. The body image and urinary incontinence scales were reliable, and construct validity was sufficient. We suggest the questionnaire to be adapted to decrease the number of individual items, improve the scales, and therefore increase reliability of the entire questionnaire.

## Introduction

Colorectal cancer is a prevalent cancer, and both the disease and its treatment strongly impact quality of life (QoL). To allow for the evaluation of new treatments, the European Organisation for Research and Treatment of Cancer (EORTC) developed the colorectal QoL module QLQ-CR38 [1] as an adjunct to the generic EORTC QLQ-C30. Later, this was revised to the shorter QLQ-CR29 [2] and validated in an international study [3]. The resulting QLQ-CR29 consisted of four scales and 19 individual items. Later validation studies were reported for the Polish [4] and Spanish [5] versions. Validation of the Danish QLQ-CR38 [6] suggested the QLQ-CR29 to be more valid than the QLQ-CR38. In the Spanish QLQ-CR29, the blood and mucus scale was not confirmed; in the Polish only the body image scale was reliable, and the urinary incontinence scale approached acceptable reliability. Construct validity was limited for the Polish version and showed ambiguous results for the Spanish. In both cases, the authors nevertheless concluded the questionnaire to be reliable and valid. These equivocal results led us to assess the reliability and validity of the Dutch version and to assess whether additional scales might result in a reduction in the number of individual items.

## Materials and methods

### Translation and procedures

The QLQ-CR29 had been translated into Flemish/Dutch by the EORTC Quality of Life Group, following their Translation Procedure Manual Instructions [7]. Differences in the Dutch language exist between Belgium and the Netherlands, and pilot testing was undertaken to reword some items for a Dutch population, in 29 patients with colorectal cancer from the Leiden University Medical Center (LUMC). Suggested changes were discussed with experts of the EORTC, resulting in a final Dutch translation.

Consecutive patients were recruited from two academic and two peripheral hospitals in the western region of the Netherlands [LUMC—Departments of Surgery and Radiotherapy, Alrijne Hospital Leiden (former locations Diaconessen Hospital and Rijnland Hospital)], and Erasmus Medical Center Rotterdam, between May 2011 and December 2012. In three departments (Diaconessen Hospital, LUMC—Surgery, and ErasmusMC), research nurses handed the questionnaire to the patients (*n* = 123, response rate 79 %) at the time of their follow-up visit, and in one hospital (Rijnland), the questionnaire was sent to patients (*n* = 80, response rate 83 %) who had undergone treatment for colorectal cancer between May and December 2011. In one department (LUMC—Radiotherapy), the questionnaire was sent to patients (*n* = 93, response rate = 78 %) who participated in other studies [8, 9]. Of the 296 patients receiving a questionnaire, 244 returned it, and we included 236 completed questionnaires (response 80 %). Time between surgery and filling out the questionnaire ranged from 5 months to 12 years. No information is available on the non-responders, unfortunately, but given the nature of the task, filling out a short questionnaire, we do not expect major non-response bias.

For convergent validity, participants were additionally asked to fill out the EORTC QLQ-C30. For test–retest reliability, we approached patients who had indicated their willingness in the first questionnaire. The questionnaire was sent to every fifth participant within 2 weeks of returning the first questionnaire. Twenty-seven patients (out of 48 invited, 56 %) filled in the questionnaire twice, on average 19 days after the first (range 4–46 days). Patient characteristics are presented in Table 1.

**Table 1:** Patient characteristics

Patients	Main sample *N* = 236	Retest sample *N* = 27
Age		
Mean age, years ± SD (range)	65 ± 11.3 (24–90)	64 ± 14.1 (24–83)
≤65 years	114 (48 %)	13 (48 %)
Male	143 (61 %)	20 (74 %)
Marital status^a^		
Single	34 (15 %)	8 (30 %)
Married	172 (75 %)	18 (67 %)
Widow(er)	24 (10 %)	1 (4 %)
Educational level^a^		
Low	74 (32 %)	7 (28 %)
Intermediate	91 (40 %)	11 (44 %)
High	64 (28 %)	7 (28 %)
Stoma, yes^b^	68 (29 %)	12 (44 %)
Curative treatment	196 (83 %)	23 (82 %)

^a^Does not count up to 236 due to missing data

^b^At the time of filling in the questionnaire

### Statistical analysis

We assessed item performance, by proportion of floor and ceiling effects, and by test–retest reliability (intraclass correlation coefficients, ICCs). Since the QLQ-CR29 was shown to consist only of few and mostly two-item scales, we carried out a principal component analysis to detect potential additional subscales, based on eigenvalues (>1.0). Items 49–54 on bowel problems (patients without a stoma) and stoma problems (patients with a stoma), respectively, were used as if the same items for patients without and with a stoma. We used varimax rotation to facilitate interpretation [10]. We assessed scale reliability using Cronbach’s *α*, for both the newly found scales and the original four scales. Subscales were constructed on the basis of the principal component analysis by adding the unweighted scores of the variables that loaded on a factor and normalizing to 0–100. Finally, we assessed construct validity as done in the earlier studies [4, 5], using correlations with the QLQ-CR30 (scores below 0.40 indicating no undue overlap between the constructs of the two questionnaires), and known-groups comparisons comparing older (≥66 years) and younger (≤65 years), patients with and without a stoma, and patients treated with curative and palliative intent using Mann–Whitney *U* tests.

## Results

### Characteristics of items

Table 2 presents the item characteristics and the subscales detected. ICCs were low for urinary incontinence and dysuria. The percentage respondents at floor was rather high (>50 %) in the blood and mucus in stool scale and for 19 individual items.

**Table 2:** Quality of life scores according the EORTC QLQ-CR29, structure and reliability

Scaling/single-item name	*n*	Item No.	Mean	SD	*α*	% floor^c^	% ceiling^c^	Range	ICCs^a^
*All patients*	236								
Urinary frequency		31, 32	32.2	24.0	0.71	22.2	1.7	0–100	0.33, 0.43
Blood and mucus in stool		38, 39	7.9	15.9	0.56	74.5	0	0–83.3	0.90, 0.72
Body image		45–47	18.5	21.7	0.80	36.0	1.3	0–100	0.76, 0.44, 0.41
Defaecation/stoma problems		49–54	21.4	19.1	0.84	9.6	0	0–88.9	see below
Urinary incontinence		33	7.6	18.2		82.3	0.9	0–100	0.20
Dysuria		34	4.1	13.7		90.2	0.4	0–100	0.36
Abdominal pain		35	11.7	22.2		73.6	2.1	0–100	0.79
Buttock pain		36	14.2	24.8		70.1	2.6	0–100	0.74
Bloated feeling		37	16.0	22.7		61.1	1.3	0–100	0.55
Dry mouth		40	18.6	25.7		58.9	2.5	0–100	0.93
Hair loss		41	8.3	20.9		83.3	2.1	0–100	0.82
Trouble with taste		42	12.3	24.9		75.7	3.4	0–100	0.75
Anxiety		43	33.8	26.4		26.3	4.2	0–100	0.54
Weight		44	20.6	26.6		55.3	3.0	0–100	0.71
*Patients without stoma*	168								
(2) Flatulence		49	34.6	27.4		27.1	3.8	0–100	0.64^b^
(2) Faecal incontinence		50	12.1	23.2		73.8	2.5	0–100	0.75^b^
(2) Sore skin around anus		51	14.7	26.8		70.8	5.0	0–100	0.82^b^
(2) Stool frequency		52, 53	24.1	22.6	0.68	30.0	0.6	0–100	0.81^b^, 0.27^b^
(2) Embarrassed by defaecation pattern		54	21.1	30.0		60.2	5.0	0–100	0.65^b^
Defaecation problems		49–54	21.8	19.6	0.84	9.9	0	0–88.9	
*Patients with stoma*	68								
(2) Flatulence		49s	30.3	23.0		28.4	0	0–66.7	See no stoma^b^
(2) Faecal incontinence/leakage		50s	20.2	26.7		57.6	1.5	0–100	
(2) Sore skin around stoma		51s	21.9	30.5		58.2	6.0	0–100	
(2) Stool frequency/bags change		52s, 53s	14.4	20.4	0.72	56.1	0	0–83.3	
(2) Embarrassed by stoma		54s	20.9	27.1		55.2	3.0	0–100	
(2) Stoma care problems		55s	8.6	19.7		81.8	0	0–66.7	
Stoma problems		49–54s	20.4	17.9	0.80	9.0	0	0–77.8	
*Male*	143								
(1) Sexual functioning		26	30.6	25.4		31.1	2.2	0–100	0.85^b^
(2) Impotence		27	41.7	41.7		42.3	26.0	0–100	0.78^b^
*Female*	93								
(1) Sexual functioning		28	16.1	22.8		61.2	1.2	0–100	See males^b^
(2) Dyspareunia		29	14.5	28.5		74.5	5.5	0–100	

^a^If two or more correlations are presented, these are in order of the items in column 3

^b^ICCs are for patients with and without stoma (defaecation) and males and females (sex) combined

^c^Percentages scoring lowest (floor) and highest (ceiling) category

### Factor analysis and reliability

Factor analysis revealed seven factors, of which the original urinary frequency scale (Cronbach’s *α* = 0.71) and body image (*α* = 0.80) scales were reproduced (alpha in the original study [3] of 0.71 and 0.84, respectively). The original two-item stool frequency scale (items 52 and 53) had a lower *α* (0.68, originally 0.70 [3]) than when included in a larger factor, with all bowel and stoma problems included (items 49–54: *α* = 0.80). This latter scale also showed good reliability for patients with (*α* = 0.80) and without (*α* = 0.84) a stoma. The blood or mucus in stool scale was reproduced in the factor analysis but had a low *α* of 0.56 (originally 0.69 [3]). All remaining factors did not form clearly interpretable scales, and reliabilities were all below 0.70. We thus present construct validation for the original scales and items, as well as the new bowel/stoma problems scale.

### Construct validity

Correlations between the subscales and the QLQ-C30 subscales were below 0.40, except for body image, which correlated moderately (*r* = 0.48) with social functioning.

Younger compared to older patients had significantly worse sexual functioning (Table 3) and had fewer problems with urinary frequency and incontinence and with a dry mouth. Patients without a stoma had a higher body image and less urinary incontinence. Patients treated with curative intent indicated more problems with blood and mucus in stool, defaecation problems, buttock pain, and stool frequency and fewer problems with hair loss and trouble with taste than patients treated with palliative intent.

**Table 3:** Known-groups comparisons (age, stoma, treatment intent)

CR29	Age			Stoma			Treatment curative intent		
	≤65	≥66	*p*	No	Yes	*p*	Yes	No	*p*
Urinary frequency	26.0 (21.4)	38.1 (25.0)	.00***	32.1 (24.6)	32.3 (22.6)	.89	32.5 (24.8)	30.8 (20.2)	.85
Blood and mucus in stool	9.2 (17.8)	6.6 (13.9)	.33	9.3 (17.6)	4.3(9.7)	.09	9.2 (17.0)	1.3 (4.4)	.00***
Body image	20.4 (23.2)	16.7 (20.1)	.24	14.0 (18.6)	29.4 (24.6)	.00***	18.6 (22.3)	17.6 (18.4)	.70
Defaecation/stoma problems	19.9 (18.7)	22.8 (19.5)	.19	21.8 (19.6)	20.4 (17.9)	.79	22.4 (19.0)	16.9 (19.4)	.05*
Urinary incontinence	4.7 (13.2)	10.5 (21.7)	.03*	5.8 (15.6)	12.1 (23.1)	.02*	7.6 (18.0)	7.7 (19.4)	.77
Dysuria	5.3 (15.7)	3.3 (11.7)	.22	3.8 (13.4)	5.1 (14.6)	.46	4.6 (14.2)	1.7 (10.5)	.10
Abdominal pain	11.7 (23.0)	11.7 (21.4)	.82	12.7 (23.3)	9.0 (18.9)	.32	11.8 (22.3)	10.8 (21.9)	.80
Buttock pain	14.2 (25.9)	14.3 (23.9)	.70	11.3 (22.7)	21.7 (28.3)	.00***	15.7 (25.4)	7.5 (20.7)	.03*
Bloated feeling	18.4 (24.7)	13.9 (20.5)	.18	17.2 (22.8)	13.4 (22.5)	.16	16.7 (23.3)	13.3 (19.7)	.50
Dry mouth	11.7 (19.8)	25.1 (28.8)	.00***	18.3 (25.7)	19.6 (25.9)	.68	17.7 (24.4)	23.3 (31.3)	.41
Hair loss	90.9 (22.4)	92.5 (19.5)	.67	8.8 (21.7)	7.1 (19.0)	.66	5.5 (15.1)	23.9 (35.0)	.00**
Trouble with taste	12.7 (24.5)	12.0 (25.4)	.66	12.0 (25.1)	13.2 (24.5)	.47	10.9 (24.5)	19.2 (26.0)	.01*
Anxiety	33.6 (27.9)	33.9 (25.0)	.79	35.5 (26.6)	29.4 (25.5)	.10	32.1 (24.9)	41.7 (31.8)	.09
Weight	18.1 (27.0)	22.9 (26.2)	.08	18.6 (26.0)	25.5 (27.7)	.04*	21.8 (26.8)	14.5 (25.1)	.07
*Without stoma*									
Flatulence	32.1 (27.3)	37.1 (27.6)	.27				36.1 (26.5)	7.6 (30.9)	.08
Faecal incontinence	12.1 (23.3)	12.1 (23.3)	1.0				13.2 (23.3)	6.9 (22.5)	.05*
Sore skin around anus	16.1 (27.9)	13.3 (25.8)	.45				14.9 (27.1)	13.8 (26.0)	.85
Stool frequency	23.9 (23.3)	24.3 (22.0)	.82				25.7 (23.1)	16.7 (18.4)	.05*
Embarrassed by defaecation pattern	16.5 (26.4)	25.8 (32.7)	.08				22.5 (30.1)	14.9 (29.0)	.15
*With stoma*									
Flatulence	28.7 (21.3)	31.6 (24.4)	.65				29.8 (23.5)	33.3 (12.1)	.62
Faecal incontinence/leakage	16.7 (24.8)	22.8 (28.1)	.36				21.8 (27.4)	12.1 (22.5)	.26
Sore skin around stoma	23.0 (32.2)	21.1 (29.4)	.91				23.2 (31.7)	15.1 (22.9)	54
Stool frequency/bags change	11.5 (20.9)	16.7 (20.0)	.12				13.9 (20.2)	1637 (22.3)	.59
Embarrassed by stoma	14.9 (22.9)	25.4 (29.4)	.15				20.8 (26.6)	21.2 (30.8)	.95
Stoma care problems	6.0 (15.9)	10.5 (22.1)	.44				9.1 (20.7)	6.1 (13.5)	.90
*Male*									
Sexual functioning^a^	37.6 (26.7)	23.1 (22.0)	.00***	31.1 (25.4)	29.4 (25.7)	.68	31.9 (25.4)	22.8 (25.0)	.14
Impotence	37.3 (42.1)	47.0 (41.1)	.21	33.3 (38.6)	61.3 (42.7)	.00***	40.9 (41.0)	47.1 (47.2)	.71
*Female*									
(1) Sexual functioning^a^	26.7 (26.4)	6.7 (13.5)	.00***	18.8 (24.6)	8.7 (15.0)	.10	16.4 (23.3)	14.6 (21.0)	.86
(2) Dyspareunia	21.0 (32.2	8.3 (23.4)	.06	9.2 (21.3)	28.9 (39.6)	.07	15.9 (30.9)	9.1 (15.6)	.89

Higher scores indicate higher levels of symptoms or less functioning

* *p* ≤ .05; ** *p* ≤ .01; *** *p* ≤ .001 (Mann–Whitney *U* test)

^a^In this area higher scores represent better functioning

## Discussion

This study largely replicates the findings of the original study [3] and the Spanish validation [5]. As in the original study, the body image and urinary frequency scales were reliable, while the blood and mucus scale was only moderately reliable. An important result is that we found a reliable scale incorporating the items about bowel problems or stoma problems. Neither the Spanish nor the Polish study performed an exploratory factor analysis and only reported the results for the scales defined in the original paper [3]. Since the original stool frequency scale was incorporated in this new scale, the questionnaire still consists of four scales, but with 14 additional single items instead of 19. For reasons of reliability and multiple testing, it is recommended to have as few single items as possible, so this is an improvement.

Remarkable was the better item performance in our study compared to the Spanish validation, where ceiling effects were present in over 50 % of the scores in four domains (body image, anxiety, weight, and impotence). The patients in our sample scored markedly lower than those in the Spanish study, likely reflecting in part cultural values about body image and sexuality. Dysuria had similar high floor effects in the Spanish [5] and Danish [6] studies. We recommend additional assessment of the items urinary incontinence and dysuria, which showed poor reliability and item performance.

Reliabilities of the items in the original study were higher than ours (ICCs > 0.55). The other studies did not report test–retest reliability.

Construct validity was sufficient, as shown by only limited overlap between the QLQ-CR29 and QLQ-C30 (similar to the original study [3], apart from the correlation only we found between body image and social functioning). We also found differences in scores between groups that were well interpretable. We found fewer differences between patients with and without a stoma than the original study [3] (which also saw differences for the urinary frequency scale and the faecal incontinence, sore skin, and embarrassment items). Further, patients receiving palliative treatment in that study reported more problems with hair loss, anxiety, faecal incontinence, and dyspareunia, whereas in our study they reported less blood and mucus in stool and buttock pain, and lower stool frequency.

In conclusion, we were able to replicate earlier findings, but could also reduce the number of single items and thus improve on the QLQ-CR29 as published so far. We recommend that the remaining individual items be revised to improve their performance, and that following that, more psychometric research be carried out to reduce the number of individual items.
